# Report of the Proceedings of the Southwestern Michigan Dental Association

**Published:** 1900-05-15

**Authors:** 


### DENTAL REGISTER.

Vol. LIV. MAY 15, 1900. No. 5.

### REPORT OF THE PROCEEDINGS OF THE SOUTHWESTERN MICHIGAN DENTAL ASSOCIATION,

HELD AT DECATUR, MICH., APRIL 10 AND 11, 1900.

The first session was called to order at 2 P. M. Tuesday, and opened with appropriate remarks by Dr. A. C. Runyan, the President, of South Haven. Owing to the absence of the first essayist on the program, the subject announced, "The Care of Children's Teeth," was presented in voluntary remarks by Dr F. H. Essig, of Dowagiac. Dr. Essig presented the subject from the standpoint of preparation for the development and eruption of a strong and regular set of permanent teeth. He also urged the importance of taking the necessary precaution to prevent the early loss of the first permanent molars. Advocated more care on the part of the profession in educating parents, as well as children, in the matter of thorough cleanliness and frequent inspection of the teeth by the dentist, so that incipient decay might be cared for before it threatened the vitality of the tooth. He also believed that public instruction should be given to the school children, either by a dentist or the teachers of hygiene, as many children could be reached by this means who would not be likely to visit a dentist until too late to be of service except to relieve pain.

### DISCUSSION.

The President called upon Dr. Hoff to continue the discussion. Dr. Hoff fully agreed with all that Dr. Essig had said; but he thought that, while much could be done in an educational way with parents and school teachers and scholars, the dental profession had not yet fully appreciated the importance of preserving the deciduous teeth. While it is true that many dentists do what is possible in this direction for various reasons, children's first teeth are sadly neglected by the majority of practitioners. Some men do not like to operate for children; some are too busy to give their time to temporary operations, and others think they can not be adequately compensated for such services; and so for many reasons the children's teeth of to-day are not as well cared for as they should be.

It is important that good and durable operations should be made on children's teeth. It is just as essential to the comfort and well-being of the child that the full contour and masticating surfaces of his teeth be maintained as that those of the adult should be conserved or restored when destroyed, nay, it is even of more importance, for the reason that if a child's teeth are not in perfect condition he will not use them for the proper mastication of his food. He will not learn to thoroughly utilize his teeth and forever looses the opportunity of forming a habit which will serve him well in the preparation of his food for the nutrition and building of vigorous tissue elements at a time when there is most need. This applies not only to the general system, but it is of great importance to the teeth themselves. If both arches are complete and there are no troublesome interproximal spaces, or sensitive teeth, the child will intuitively and unconsciously use the teeth more uniformly and effectively. The result of this exercise will be noted afterward in the development of strong and well developed permanent teeth, with an adequate development of the jaws and other tissues for their support and nourishment And the habit of thorough mastication in childhood will be easily carried forward to youth and manhood I fully realize that it is impracticable to prevent carious destruction, or attacks at least, of the deciduous teeth, but I believe. by adequate supervision, this could be reduced to the minimum in the majority of cases that could be kept under adequate supervision And where decay occurs and the proximal surfaces are destroyed breaking the continuity of grinding surface the best possible efforts are none too good and should be attempted to restore these lesions to a normal condition.

This brings up another and larger subject which I do not care to discuss, now at least, namely, the kind of filling material to use in children's teeth. In my own hands for crown and proximal cavities which do not involve the proximal and occlusal angles I find the cements, gutta-percha and tin foil valuable and effective. In large compound cavities I use amalgam, or what I like better, amalgam and cement mixed together, and the surfaces and angles contoured with a veneer of amalgam, according to the method suggested by Dr. H. A. Knight, of Minneapolis.

Dr. W. E. Harper: Advocated giving instruction in schools in oral hygiene. Would begin with the little ones of the kindergarten departments and make the use of the tooth brush a part of the instruction. It is greatly to be desired that the deciduous teeth receive attention before pulp complications are reached, because these conditions render conservative operations frequently impracticable. It is many times difficult to make operations for children when brought to the dentist. I make it a point to educate children to endure operations gracefully. I am careful never to inflict pain at the first few sittings, but gradually lead up to the operation which necessarily involves pain infliction. By some coddling and much patience it is possible to cultivate a spoiled or refractory child into a well-behaved patient. As a rule, I do not care to have an officious nurse or even the child's mother present when I am operating for a child. I almost never use the dental engine on children's teeth. By properly selected and using hand instruments it is practicable to prepare in a short time any cavity in a child's tooth so that it can be well filled with the plastic filling materials. I make proximal and restoration operations with amalgam.

In regard to the first permanent molar, I extract it when badly decayed, and especially when complicated with a putrescent pulp, and especially if I get the patient before twelve years of age.

Dr. C. J. Siddell: I find it impracticable to use hand instruments entirely in the preparation of some cavities in children's teeth. It involves the application of so much force to cleve the enamel, oftentimes necessary in the proper formation of a cavity, that the child will seriously object In such cases I think the engine much preferable.

I have used a filling material for children's teeth of gutta-percha into which had been incorporated dry powdered Portland cement It makes a hard filling which seals the cavity perfectly and stands the wear well.

I believe it good practice to extract the first permanent molars when decayed, if it can be done at any time prior to the emergence of the second permanent molar.

Dr. S. M. White: If we can take care of the teeth of the children and youth, we have taken the best method for saving the teeth of the adults We should have a dentist as a member of every Board of Health It should be made compulsory upon all children to have their teeth periodically examined by a competent dentist who should report to the Board of Health or the child's parents the condition of his teeth and recommend a course of treatment

Dr. C B Roe: The principal of the schools in my town a few years ago invited me to give a talk to the children of the schools. I did so and have since continued it, even to the extent of carrying on a regular class exercise. I have the children make copies of teeth which I give them or from drawings put before them. I have found the work full of interest and think it has been profitable

Dr T. G Rix: It is a difficult matter to attempt any kind of public professional education in a town where there is more than one dentist. The other practitioners will discredit your efforts, and even the dear people will misconstrüe your motives. I should not like to attempt it in my own town, and I do not think my confrees are capable of doing the subject justice (laughter). My experience is, that we shall have to do this work in our own offices

Dr S M. Fowler: I should like to have some one tell me how I can get the public to come to my office I think I will do the talking if I can get the chance. It has often occurred to me that some good might come from reading papers on dental hygiene at Teachers' Institutes, and in this way reaching the public school teachers who have many opportunities to impress the facts of dental and oral hygiene upon their pupils

Dr. E. A. Honey: Several years ago the State Dental Association made an effort to secure legislation looking toward a systematic investigation of the condition of the teeth of the school children Just what came of the effort I have never learned It seems as though there should be a public charitable hospital in every community where the poor children, at least, might secure needed service without cost.

Dr. Honey suggested that this society appoint a committee, to co-operate with a similar committee from the

State Association, which should appeal to the State Board of Health to inaugurate a campaign for creating public sentiment along this line, if nothing further.

DR. A. C. RUNYAN: An effort of this kind is now being made in Illinois, and it is hoped that good will result from it.

In regard to reading addresses at Teachers' Institutes, I would say that for several years I have been invited to deliver a short course of lectures before a summer school. I have made use of models and casts to illustrate the function of the teeth, and various common diseases to which the teeth are liable. The interest manifested has been sufficient to encourage me to do the work as well as I could, and I know it has been appreciated. I have specially called attention to the fact that the first permanent molar should not be neglected until too far decayed to be saved. I regard the loss of this tooth as a great misfortune, as it will certainly cause a readjustment of the positions of the other permanent teeth which too often is so faulty as to make a bad occlusion and an imperfect masticating surface

DR. HARPER: The extraction of the first lower permanent molars is a great mistake, as it is certain to produce an unnatural occlusion It is not so bad when all four of the first permanent molars are extracted at the same time, or at least at an early period of the child's life.

I also want to say that it is not necessary to exert much force when using hand instruments in the preparation of cavities in children's teeth. The instruments should be sharp and adapted to the purpose By observing the rules governing cavity preparation: cleavage of enamel, outline, retention angles, etc, it does not require much force or pressure nor more time than would be consumed in doing the work with the dental engine.

In extracting the deciduous teeth I make it a point not to extract the second deciduous molar until the second permanent bicuspid is ready to take its place. It is not so important to save the first deciduous molar.

Dr. J. A. Watling: The extraction of the first molars, where badly decayed, I regard as an excellent practice. If the lower molars are extracted between the ages of ten and eleven and the uppers between twelve and fifteen years, the occlusion will be good and the teeth will stand perpendicular. A molar with a dead pulp is not likely to last more than ten years, and its loss at such a time will create a much greater deformity in the occlusion of the teeth than could possibly occur were it extracted at an earlier date and no attempt made to save it.

In regard to a filling material for children's teeth I make much use of gutta-percha. Where the teeth are decayed on adjoining proximal surfaces I often fill both cavities at the same time without separation. thus bridging over the interproximal space and preserving a continuous masticating surface until the time arrives for the displacement of the deciduous teeth by the permanent. Properly adapted gutta-percha fillings made in this way will last in most mouths for five or six years.

The subject was then passed, and the Secretary read, in the absence of its author, a paper written by Dr. W. V. B. Ames, of Chicago, on "Oxyphosphates."

